# Building a Better Fragment Library for *De Novo* Protein Structure Prediction

**DOI:** 10.1371/journal.pone.0123998

**Published:** 2015-04-22

**Authors:** Saulo H. P. de Oliveira, Jiye Shi, Charlotte M. Deane

**Affiliations:** 1 Department of Statistics, Oxford University, Oxford, Oxfordshire, United Kingdom; 2 Department of Informatics, UCB Pharma, Slough, United Kingdom; 3 Shanghai Institute of Applied Physics, Chinese Academy of Sciences, Shanghai, China; University of Michigan, UNITED STATES

## Abstract

Fragment-based approaches are the current standard for *de novo* protein structure prediction. These approaches rely on accurate and reliable fragment libraries to generate good structural models. In this work, we describe a novel method for structure fragment library generation and its application in fragment-based *de novo* protein structure prediction. The importance of correct testing procedures in assessing the quality of fragment libraries is demonstrated. In particular, the exclusion of homologs to the target from the libraries to correctly simulate a *de novo* protein structure prediction scenario, something which surprisingly is not always done. We demonstrate that fragments presenting different predominant predicted secondary structures should be treated differently during the fragment library generation step and that exhaustive and random search strategies should both be used. This information was used to develop a novel method, Flib. On a validation set of 41 structurally diverse proteins, Flib libraries presents both a higher precision and coverage than two of the state-of-the-art methods, NNMake and HHFrag. Flib also achieves better precision and coverage on the set of 275 protein domains used in the two previous experiments of the the Critical Assessment of Structure Prediction (CASP9 and CASP10). We compared Flib libraries against NNMake libraries in a structure prediction context. Of the 13 cases in which a correct answer was generated, Flib models were more accurate than NNMake models for 10. “Flib is available for download at: http://www.stats.ox.ac.uk/research/proteins/resources”.

## Introduction

Fragment-based *de novo* protein structure prediction is the current standard for template-free modelling of proteins. This approach, exemplified by ROSETTA [1,2,3,4], relies on creating a library of fragments extracted from known protein structures. In this context, a structural fragment is a continuous subset of the residues of a protein. Such structural fragments are usually less than 20 residues long. Each fragment in the library represents a specific position in the sequence to be modelled (target). The most common technique used to select library fragments is the sequence similarity between the fragment and the region of the target sequence that the fragment represents. Fragments from the library are pieced together in order to generate complete models of the target structure. There are many fragments in the library for each position in the target (e.g. ROSETTA's fragment libraries contain 200 fragments per position [1]) and many proteins are hundreds of residues long. In order to explore this large combinatorial space, heuristics are needed [4]. Commonly used heuristics rely on statistical and physical potentials to ensure that global structural features of proteins are sustained/respected in the models generated from the combinations of fragments.

A major problem for all fragment-based *de novo* approaches occurs when the fragment library for a given target does not contain good fragments for a particular region. In that case, low accuracy models will be generated regardless of the precision of the potentials being used and regardless of the amount of computation time invested in the modelling routine [5]. For that reason, accurate fragment library generation is crucial to the success of template-free modelling [5].

NNMake [6] is ROSETTA’s method for fragment library generation. NNMake extracts fragments from a template database of non-identical high resolution structures (<2.5 Å). It scores every length nine segment of the target sequence exhaustively against all length nine segments within its template database. NNMake's score is based on sequence similarity, predicted secondary structure and predicted torsion angle. A library for the target sequence consists of the 200 top-scoring fragments per target position. The latest version of NNMake was tested on a set of 62 small globular proteins (all shorter than 150 residues in length). In their test procedure, the authors removed all sequence homologs. In order to assess the quality of generated fragment libraries, ROSETTA was used to generate decoys for each target. The number of decoys generated varied according to target length (ranging from 4,065 to 19,183 decoys). The average coordinate root-mean square deviation (cRMSD) between the native structure of the target and the top 0.1 percentile models were computed (average of the 62 targets cRMSD = 3.75 Å). One of the current limitations of NNMake relates to the use of fragment with a fixed length (nine residues long). This may not be ideal as it has been shown that accurate structures can be built using fragments as short as four residues [7]. It has also been reported that fragments longer than nine residues can generate better results if the modelling routine is adjusted accordingly [8]. Other fragment generation software extracts either longer fragments or fragments with varying lengths [9, 10, 11, 12].

Other fragment library generation software also attempt to increase the amount of local structural information used to generate libraries. For example, FRAGFOLD [13] uses super-secondary structure fragments, which express the relationship between consecutive secondary structure elements. FRazor [14] builds on NNMake and incorporates solvent accessibility and contact capacity into its scoring scheme. The authors of FRazor claim that their scoring scheme improves the precision of NNMake libraries by discarding low quality fragments suggesting that a sequence-based score can benefit from additional structural information.

HHFrag [10] selects fragments slightly differently from many other methods: by means of profile hidden markov models (HMM). HHFrag uses the HHpred toolchain [11] to build a profile-HMM of the target sequence and a profile-HMM for each sequence in a pre-defined template database. The template database used by HHFrag is the April, 2010 build of PDBselect25 [15], a subset of 4,824 protein chains with less than 25% identity extracted from the PDB [16]. Sequence and predicted secondary structure information are used in the generation of the profile-HMM. The HMM of the target is divided into a set of overlapping HMM fragments of variable length (6−21 residues). Fragment extraction is performed by HMM-HMM alignment using the HHSearch algorithm [17]. Each of the 6 to 21-long target HMMs is aligned and scored against every HMM profile for the proteins in the template database. All fragments with a probability ≥ 0.2 are accepted. For positions where a minimum of ten fragments are not identified, fragments with lower probabilities are accepted, if possible, until the minimum threshold of ten fragments is fulfilled. HHFrag was tested on a set of 105 proteins [18]. The average length of fragments obtained was 10.3 ± 3.6 residues. In order to assess fragment library quality, two measures were defined: precision and coverage. Precision is defined as the proportion of good fragments (RMSD to native structure < 1.5Å) in the library. Coverage is the percentage of target residues represented by at least one good fragment in the library. HHFrag reports a higher precision (62 ± 16%) compared to NNMake (38 ± 17%). However, sequence homologs were not excluded from the HHFrag results, which may inflate the method’s precision. HHfrag also reports a coverage of 71 ± 13%, which is far lower than NNMake (~92%). For some target positions, HHFrag does not output any fragments, which can cause difficulties during the modelling step.

A recent fragment library generation programme SAFrag [19] also builds HMM profiles to detect fragment candidates, in an analogous fashion to HHFrag. SAFrag HMM profiles are extrapolated from a 27 state structural alphabet. The extracted fragments vary in length from 6 to 27 amino acids. Fragments are scored based on a profile-profile comparison, using the Jensen Shannon divergence. Two different template databases can be used by SAFrag: pdb25 and pdb50. Pdb25 is the same database used by HHFrag, whereas pdb50 imposes a 50% pairwise sequence identity cutoff. The method was validated on a set of 111 targets [18]. SAFrag reports a higher precision and coverage than HHFrag. However their cutoff for defining a good fragment is less strict (RMSD to native structure < 2.0Å). They also allowed homologs and the target structure itself to be included in their template database. Further, the method outputs on average less than two fragments per target position, which suggests that homolog structures are dominating the fragment libraries generated by SAFrag. SAFrag is only available as a web-server.

As described above, different methods diverge in the number of fragments used per position, in the length of the fragments used, in the selectivity of the template databases from which fragments are extracted, and in the way the extraction is performed. In this work, we investigate these aspects of fragment library generation and how they affect the precision of the library. We also evaluate the impact of including/excluding homologs among the set of known structures that fragments can be extracted to assess its impact on methods such as HHFrag and SAFrag. In a real *de novo* structure prediction method homologs would not be available, so it is important to exclude those fragments during method training and validation.

Current methods score all types of fragments using the same methodology regardless of the predominant predicted secondary structure of the fragment. Here we analysed the relationship between the predominant predicted secondary structure of fragments and our ability to accurately predict fragments for that position. We observed that fragments with a predominant predicted secondary structure (e.g. α- helical fragments) can be predicted more accurately than other types of fragments.

Based on these analyses, we have implemented Flib, a fragment library generation software that exploits the predominant predicted secondary structure of fragments to increase the precision of generated fragment libraries. We have generated fragment libraries for two validation sets: a set of 41 structurally diverse proteins extracted from the PDB (PDB-representative set) and a set of 275 protein domains that were used in CASP9 [20] and CASP10 [21] (CASP set). Fragment libraries generated by Flib were compared with libraries generated by NNMake [6] and HHFrag [10] and found to obtain the best balance between precision and coverage in both test sets.

Finally, we used the Flib libraries for protein structure prediction, using our custom implementation of a fragment-based protein structure prediction algorithm, SAINT2 (based on [22]). We were capable of generating accurate (TM-Score to native structure > 0.5) predictions for 12 of the 41 proteins in our test set. We compared our modelling results against running SAINT2 with NNMake fragment libraries. NNMake libraries generated accurate models for 8 cases. Of the 13 cases for which accurate models were generated using SAINT2, Flib libraries generated more accurate models in 10 cases.

These results indicate that Flib can be used to improve the accuracy of *de novo* protein structure prediction and demonstrate the importance of discriminating between different secondary structure fragments when performing fragment extraction.

## Results

### Fragment Library Quality Assessment

We assess library quality using two commonly employed metrics: global precision and coverage. Precision is defined as the number of good fragments divided by the total number of fragments in a library (the proportion of good fragments in the libraries). Coverage is defined as the number of residues represented by at least one good fragment divided by the number of residues of the target (the proportion of protein residues represented by a good fragment). Different methods employ different cutoffs to distinguish between good fragment conformations and bad fragment conformations. Instead of selecting a single cutoff, we have varied the good fragment cutoff between 0.1 to 2.0 Å computing the precision and coverage across the range.

### The Rationale Behind Flib

**Fig 1:**
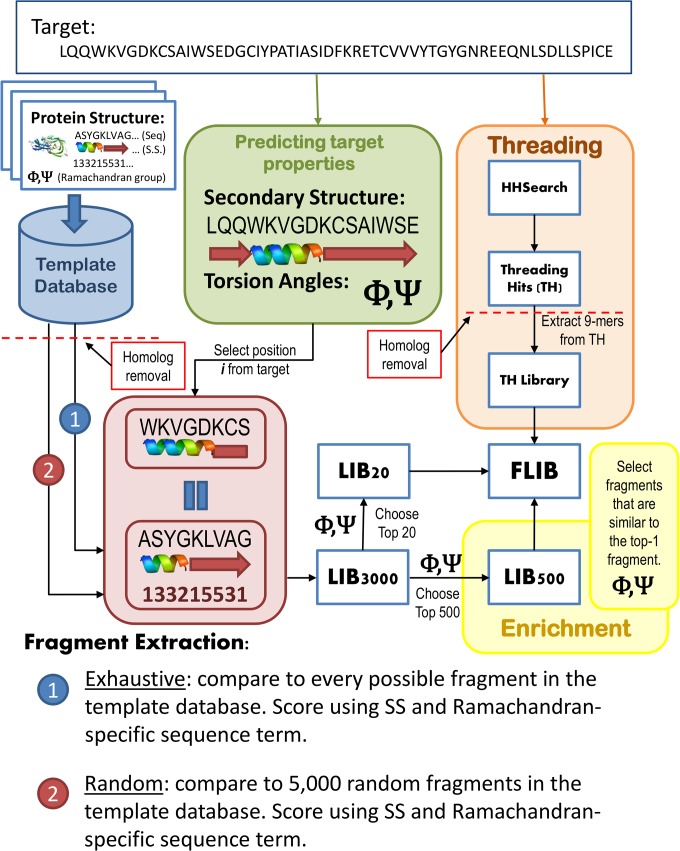
Schematics of Flib. Starting from a target sequence, we predict secondary structure (SS) and torsion angles for the target (green). We extract fragments from a template database using a combination of random and exhaustive approaches. Fragments are extracted for each target position. A library containing the top-3000 fragments per position is compiled using the SS score and the Ramachandran-specific sequence score (LIB3000). LIB3000 is then sorted according to the torsion angle score and the top-20 fragments per position are selected to comprise the final library. The final library (FLIB) is complemented by fragments that originate by an enrichment routine (in yellow) and fragments that originate from protein threading hits (orange).

Flib extracts fragments from a database of known structures using a target sequence. A framework describing Flib’s pipeline is shown in Fig 1.

### Template Database Construction

The template database, the initial set of structures from which fragments for libraries will be extracted, can be built in many ways. Regardless of how the template database is built, for testing purposes it is important to remove homologs to the target. We use the Flib template database, in which we impose a 90% sequence identity cutoff and a 5.0Å resolution cutoff, as it proved to lead to the most accurate fragment libraries (see S1 Fig).

### Fragment Extraction

The next step in a fragment library generation pipeline is fragment extraction. Most fragment library generation software methods use an exhaustive search approach [6, 7, 8, 10, 19]. We compared the use of an exhaustive approach and a random approach. In our exhaustive approach, every fragment ranging from 6 to 20 residues in the template database is scored against all of the positions of the target. The exhaustive library is composed of the top 1,000 scoring fragments per target position. The score used in Flib is based on a sequence component, a predicted secondary structure component and a predicted torsion angle component (see Methods section for more details).

**Fig 2:**
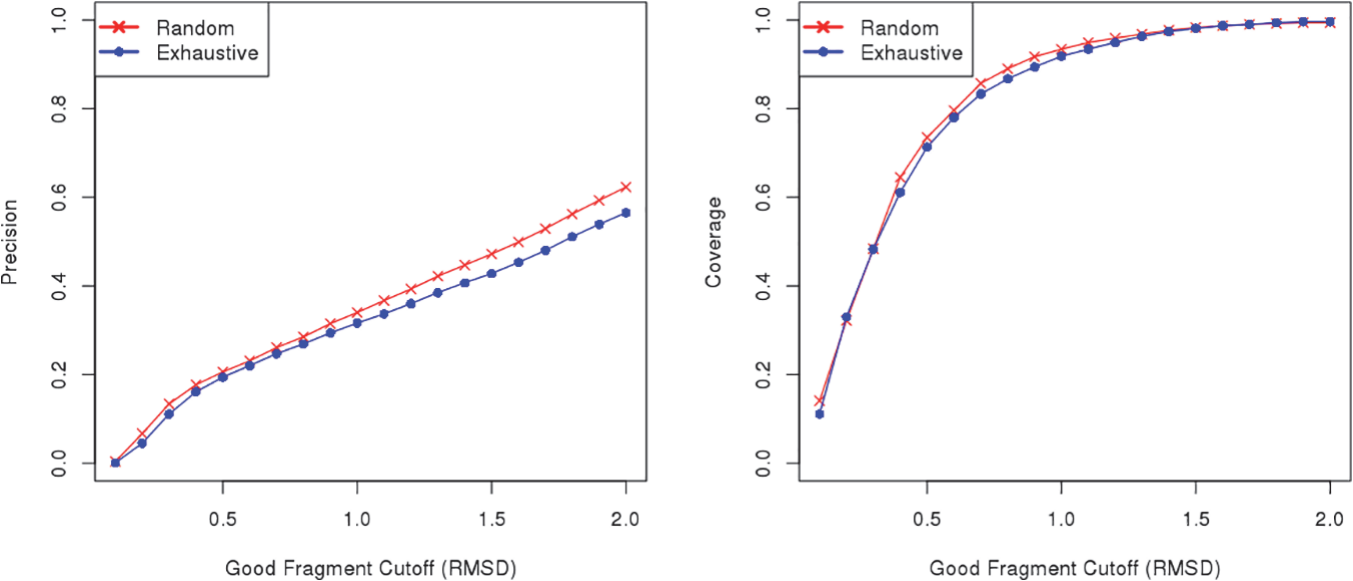
Comparison between Flib’s random extraction and exhaustive extraction methods. Analysis of the precision of fragment libraries generated by Flib using two different approaches for fragment extraction: random extraction (red), and exhaustive extraction (blue). We varied the RMSD to native structure cutoff to define a good fragment from 0.1 to 2.0 Angstroms (x-axis). The average precision on the 43 proteins in the test data set (left) and the average coverage (right) are shown for fragment libraries containing the top-1000 scoring fragments extracted exhaustively or at random. The precision indicates the proportion of good fragments in the generated libraries (y-axis).

We also compared our exhaustive approach to a random approach, in which 5,000 randomly selected fragments are scored for each target position. Fragments that satisfy a predetermined score cutoff are accepted and fragments that do not are discarded (see Methods for more details). On average, the random library contains 2,000 fragments per position. Surprisingly, we observed that fragment libraries extracted at random present slightly higher precision and coverage than the ones generated exhaustively (Fig 2). This is probably because above a certain score threshold, we observe no correlation between the scores and fragment quality (S2 Fig).

When comparing our three scores (sequence, secondary structure and torsion angle scores) we observed a higher correlation between predicted torsion angle score and fragment RMSD to native structure compared to the other two (S2 Fig). This indicates that the predicted torsion angle score is better suited to rank the fragments in the final ensemble. Within Flib’s pipeline, we combine the exhaustive and random libraries. This combined library has, on average, 3,000 fragments per target position (LIB 3000). We rank all the fragments in this library and output the 20 top-scoring fragments per target position according to the torsion angle score (LIB20). On average, within our test data set, 69% of the fragments in LIB20 are extracted by the random method and 31% by the exhaustive protocol.

### Enrichment Step

We observed that the ensemble with the highest scoring fragment per position according to the torsion angle score presented a very high precision, albeit at a loss of coverage. We decided to exploit this by implementing an enrichment step, in which we include fragments from LIB500 (analogous to LIB20, but considering the 500 top-scoring fragments) that present less than 0.5Å RMSD to the highest scoring fragment according to the torsion angle score. On average, 6.5 fragments per position are added to LIB20 during the enrichment step.

### Protein Threading Hits Library (TH Library)

**Fig 3:**
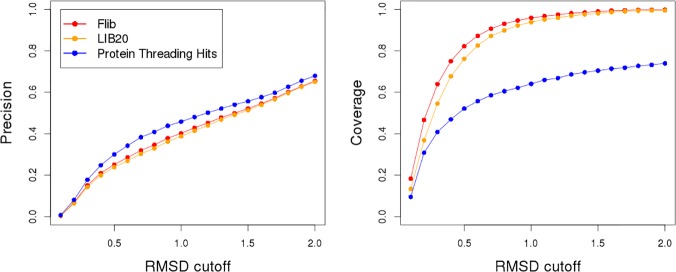
Effect of protein threading hits on fragment library quality. Analysis of the impact of fragments extracted from protein threading hits. Precision and coverage are shown for the fragment libraries generated by LIB20, Protein Threading Hits and Flib (a combination of the other two approaches). We varied the RMSD to native structure cutoff to define a good fragment from 0.1 to 2.0 Angstroms (x-axis). The average precision and coverage on the 43 proteins in the test data set is shown for each approach. The precision indicates the proportion of good fragments in the generated libraries (y-axis). The coverage indicate the proportion of residues of the target represented by at least one good fragment.

The final step in our fragment library generation routine is to add to LIB20 fragments extracted from protein threading hits. Protein threading identifies protein segments that present structural similarity to a given target. As we remove homologs, these protein threading hits are too unreliable to be used as templates for template-based modelling, but may still provide locally similar fragments. We have found that adding such fragments (on average, less than five fragments per position), when they are available, to LIB20 increases the precision of our method (Fig 3).

### Predominant Predicted Secondary Structure Determines Fragment Quality

Secondary structures (α-helices and β-strands) have restrictions by definition in the torsion angles of their residues, whilst loop regions are not so constrained and can assume a wider range of conformations [23]. Hence, secondary structure elements have a lower degree of conformational variability.

Considering that fragments with a larger number of loop residues will present a higher variability, we hypothesized that they would be harder to predict. In order to test our hypothesis, we have investigated the relationship between the RMSD to the native structure and fragments with different predominant predicted secondary structures.

Here a fragment is described as representing a target position N when it represents all the target residues between N and N+L (where L is the length of the fragment). We classify a target position as belonging to one of four distinct classes based on the predominant predicted secondary structure of its residues. The four classes of secondary structure (SS classes) are: *majority α-helical*, *majority β-strand*, *majority loop* and *other* (no predominant predicted secondary structure).

**Fig 4:**
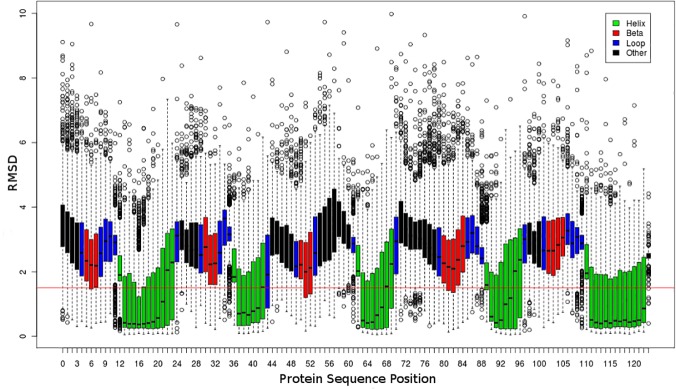
Relationship between secondary structure class (SS-Class) and fragment quality. Boxplot of the RMSD to native structure (y-axis) of 200 fragments per target position (x-axis) for the protein 1E6K. The top-200 scoring fragments from its LIB3000 were selected and are displayed. This subset of LIB3000 was chosen to increase performance of data visualization. Four Different SS Classes are defined: *majority α-helical* (green), *majority β-strand* (red), *majority loop* (blue) and *other* (black). Positions for which fragments are *majority α-helical* or *majority β-strand* present significantly lower RMSDs to the native structure and a smaller spread compared to *majority loop* and *other* positions.

We analysed the RMSD to the native structure of fragments extracted at random. The fragments were grouped according to our predicted SS classes. The spread of fragment RMSD for the top 200 scoring fragments is shown for every position in the target 1E6K (Fig 4). THE RMSD spread of the top-200 fragments replicate the results obtained with LIB3000. This figure typifies our general observation that that there is a strong relationship between the RMSD to the native structure and our four SS classes. The RMSDs for *majority α-helical* fragments are significantly lower than the RMSDs for other SS classes. *Majority loop* fragments and *other* fragments show a wider variability and are much harder to predict accurately. The difference between SS classes is important in two ways. Firstly as current methods only offer coverage and precision across all SS classes, very poor *majority loop* and *other* precision may be hidden by high *majority α-helical* precision. Secondly, these results suggest that during fragment library generation, it may improve results if we treat fragments differently according to their predominant predicted secondary structure. For that reason, Flib uses different cutoffs for accepting fragments based on SS class. Less stringent cutoffs for majority loop and other fragments are used as their variability is far higher. We have observed that adopting different cutoffs for each SS class improves the precision of the libraries generated by Flib (S3 Fig).

The usefulness of the fragments added at the protein threading step also differs between each SS classes. Adding the threading fragments to LIB20 increases the precision for *majority β-strand*, *majority loop* and *other* SS classes, but decreases the precision for *majority α-helical* fragments (S4 Fig). Thus, no fragments from threading hits are added to the *majority α-helical* target positions in our final library.

### Cross-comparison between Flib and other software

**Fig 5:**
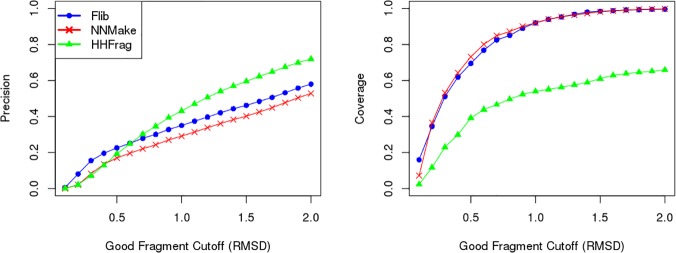
Comparison between HHFrag, NNMake and Flib. Precision (left) and coverage (right) of fragment libraries generated using NNMake (red), HHFrag (green) and Flib (blue). The precision and coverage of the fragment libraries are averaged on a set of 41 structurally diverse proteins. We varied the RMSD cutoff to define a good fragment (x axis) and evaluated the precision (proportion of good Fragments in the libraries) and coverage (proportion of protein residues represented by a good fragment) for each method.

**Fig 6:**
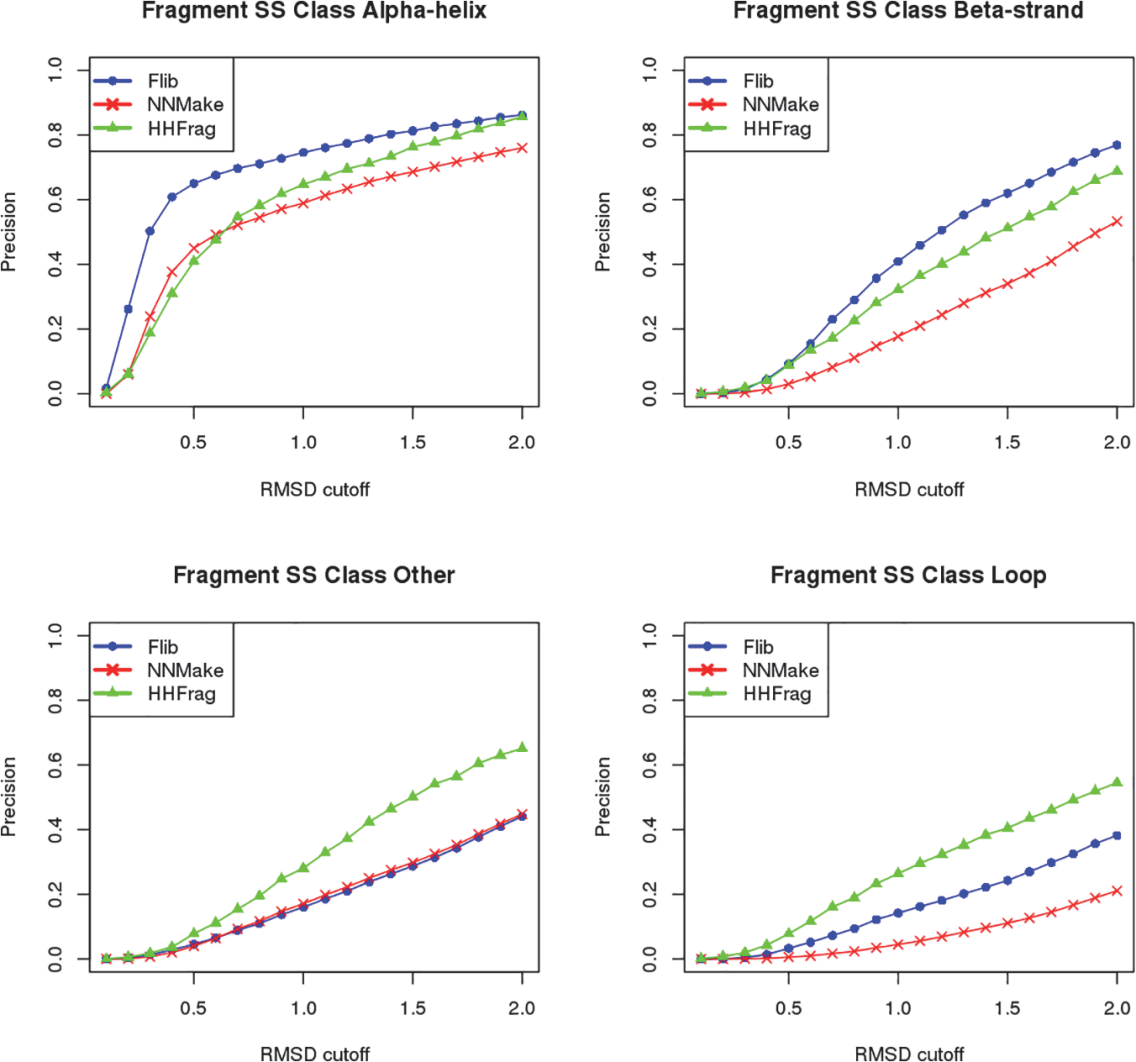
Comparison between HHFrag, NNMake and Flib. Precision of fragment libraries generated using NNMake (red), HHFrag (green), and Flib (blue) separated by SS Class. The precision of the fragment libraries were averaged on a set of 41 structurally diverse proteins. We varied the cutoff to define a good fragment (x axis) and evaluated the precision (proportion of good fragments in the libraries) for each method within four different SS classes: majority α-helical (top left), majority β-strand (top right), majority loop (bottom right) and other (bottom left).

We have compared Flib against NNMake and HHFrag (Figs 5 and 6). A large scale comparison including SAFrag could not be performed because the software is only available as a web-server. In order to perform a rigorous comparison, we have used two distinct validation sets: a set comprised of 275 protein domains from CASP9 and CASP10 (CASP set) and a set of 41 structurally diverse proteins (PDB-representative set). The second set was built to be representative of the PDB, both in terms of protein lengths and distribution of proteins amongst different SCOP classes.

In all analyses, fragments extracted from homologs were discarded (the impact of filtering out fragments from homologs is described in the next section). If we compare the overall precision and coverage, Flib presents higher precision compared to NNMake and higher coverage compared to HHFrag (Fig 5). This difference appears to be due to the increase in performance for the SS Classes majority α-helical and majority β-strand (Fig 6). HHFrag coverage is significantly lower than that of the other two methods. At a 1.0Å RMSD cutoff, HHFrag's fragment libraries describe slightly more than half of the target residues correctly (~55% coverage on PDB-representative set, ~65% coverage on the CASP set). HHFrag failed to produce any fragments for ~13% of the positions. This can become a problem during structure prediction considering that modelling routines generally require at least one fragment representing every target position.

When comparing the three programmes, Flib achieves the best balance between coverage and precision. Considering a good fragment cutoff of 1.0Å, HHFrag presents the highest average overall precision, ~43%, compared to Flib, ~35%, and NNMake, ~29.1% (data shown for the PDB-Representative set). But HHFrag's precision is increased due to a reduced number of fragments output per position (see below). HHFrag also boosts its precision by not outputting any fragments for regions that are harder to predict (as stated above, on average, ~13% of the residues are not represented by any fragment in an HHFrag generated library). Not outputting fragments for low confidence regions will improve precision, but will also cause difficulties during protein structure prediction. Data for the CASP set can be found in S5 Fig.

On the PDB-representative validation set, Flib outputs, on average, 26 fragments per position, with an average length of ~7.4 residues. HHFrag outputs on average 10 fragments per position, with an average length of 9.1 residues. Generating a smaller number of fragments can improve precision, but can represent a problem during modelling since less conformations will be sampled. NNMake always outputs 200 fragments per position with a constant length of nine residues.

The three methods perform well at predicting fragments for α-helical segments, however, at 1.0Å RMSD cutoff, Flib's precision is 74.6%, which is higher than NNMake's 59% and HHFrag's 64.7% (data shown for the PDB-representative set). Flib's precision for β-strand fragments is also higher. At 1.0Å RMSD, Flib presents 41% precision against NNMake's 17.7% and HHFrag's 32.2% (data shown for the PDB-representative set). The precision of Flib for the other two SS classes are comparable to NNMake’s precision (less than 5% difference in precision), but lower than HHFrag's. The coverage of Flib libraries slightly exceeds the coverage of NNMake for all SS classes. Results for the CASP set are included in S6 Fig.

To assess the statistical significance of our results, we have compared the distribution of RMSDs to the native structure of all fragments output by Flib and NNMake, for all targets in our PDB-representative validation set (S7 Fig). We performed a Kolmogorov-Smirnov test (alternative hypothesis that the cumulative distribution function of the RMSDs of Flib fragments is greater than the cumulative distribution function of the RMSDs of NNMake fragments) and we obtained a p-value of 2.2e^-16^. This indicates that Flib generate fragments with statistically significant lower RMSDs compared to NNMake.

### Effect of Homologs on Fragment Library Quality

We carried out an analysis to assess the impact of extracting fragments from sequence homologs of the target protein on fragment library quality. It has been shown that when a suitable template (a homolog) can be found for a specific target, template-based modelling is the most accurate way to model the structure of that target [21]. Hence, *de novo* protein structure prediction tends to be used only in cases where no homologs can be found. For that reason, a fragment library that is representative of a real *de novo* protein structure prediction case should not contain fragments extracted from homologs. Nonetheless, not all current methods for fragment library generation exclude such fragments from their outputs or from their published tests [e.g. 6, 10, 13, 19].

**Fig 7:**
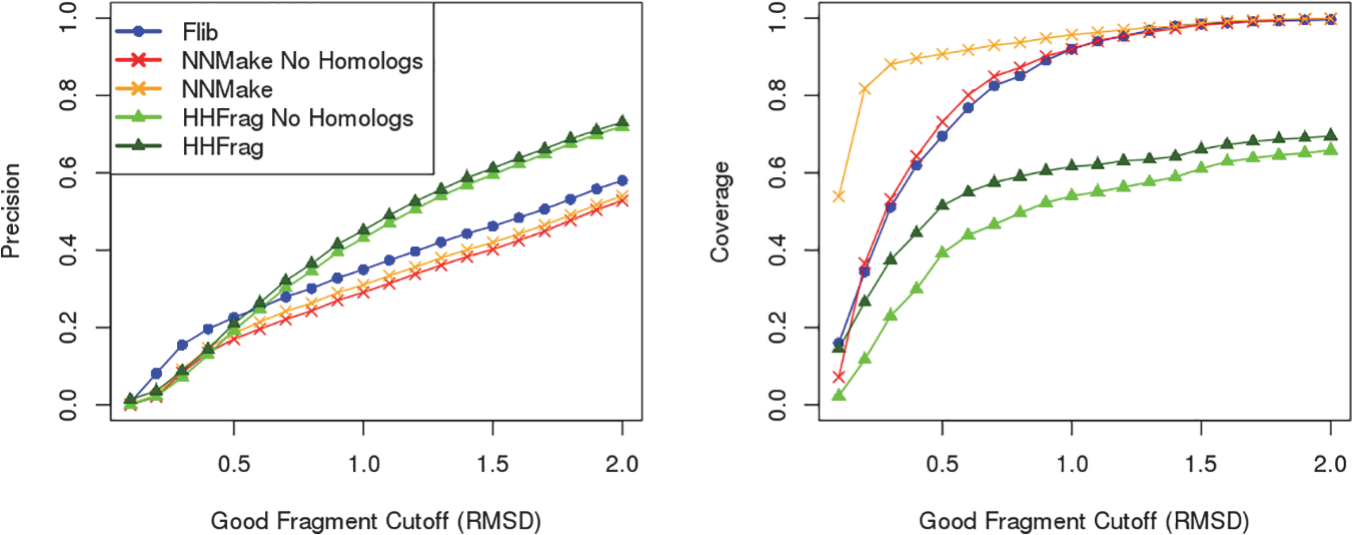
Effect of Homologs on fragment library quality. Precision (left) and coverage (right) of fragment libraries generated using three different methods: Rosetta’s NNMake (crosses), our method Flib (circles), and HHFrag (triangles). We varied the cutoff to define a good fragment (x axis) and evaluated the precision (proportion of good fragments in the libraries) and coverage (proportion of protein residues represented by a good fragment) for each of the methods when: homologs are included (red and orange) and when homologs are excluded (light and dark green). Homologs are always excluded from Flib (blue).

We have analysed the impact of including/excluding fragments extracted from sequence homologs from fragment libraries generated by two different methods: NNMake [6] and HHFrag [10] (Fig 7). Homologs were extracted from the significant hits output by HHSearch (see Methods section for detail). Results were generated using the PDB-representative set of 41 structurally diverse proteins of lengths varying between 60–500 residues (see S1 Table). HHFrag does not provide an option to exclude homologs from their template databases, and fragments resulting from homologs had to be removed from their final outputs in a post-processing step.

We have compared the precision and coverage of fragment libraries generated before and after homolog removal. If we consider the cutoff of 1.0Å to define a good fragment, homolog exclusion leads to a loss of precision from ~35% to ~29.1% for NNMake and from ~45% to 43.1% for HHFrag libraries. Homolog exclusion leads to a loss of coverage from ~61.6% to ~54% for HHFrag libraries and from ~96% to 92% for NNMake libraries. Fragments extracted from homologs increase the precision and coverage of fragment libraries.

### Model Generation/Protein Structure Prediction

Our results indicate that Flib libraries present higher precision and coverage when compared to NNMake’s. However, in order to determine how well those results translate to protein structure prediction, it is necessary to test the applicability of our libraries within a protein modelling framework. Therefore, we have generated models using Flib libraries first to assess if accurate models could be generated using those libraries and second to compare to the models generated using NNMake’s libraries. We used our custom implementation of the fragment-based *de novo* structure prediction software, SAINT2, to combine the fragments and to sample the conformational space (see Methods for more details).

**Fig 8:**
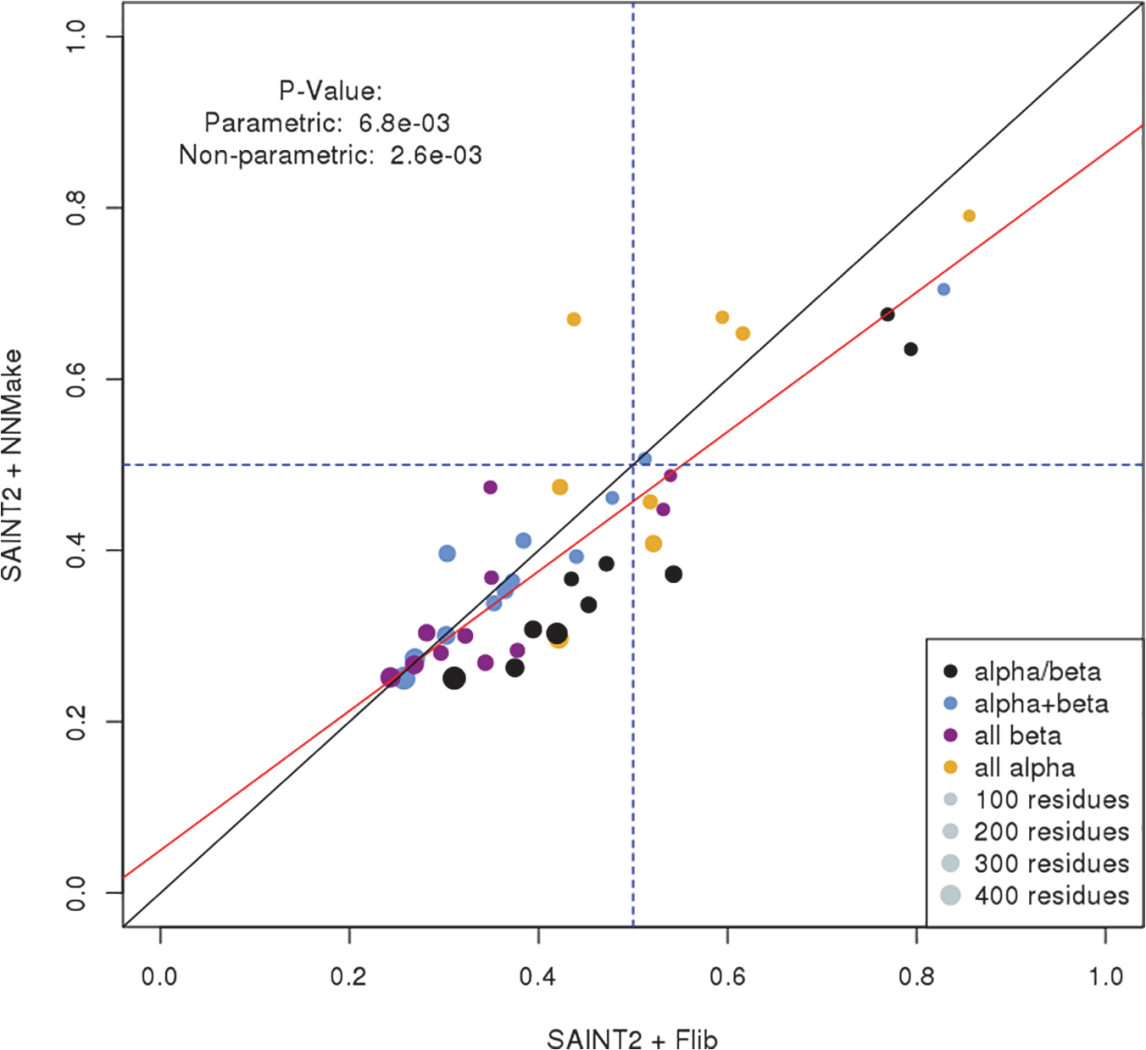
TM-Score of the best decoy as generated by Flib+SAINT2 and by NNMake +SAINT2. For each approach, 1,000 decoys were generated and the best decoy (highest TM-Score when superimposed to native structure) was chosen. Results are shown for the 41 proteins in our data set. We compared the TM-Score of best decoy generated by Flib + SAINT2 (x-axis) against NNMake + SAINT2. Each point represents a target. Point color represents the target's SCOP class and the point size is proportional to the protein length. The dotted lines indicate the cutoff for defining an accurate model (TM-Score > 0.5). Flib libraries generated accurate models for 12 of the 41 cases in our PDB-representative set. NNMake libraries generated an accurate model for 8 of the 41 cases. On the 13 cases for which accurate models were generated, Flib libraries performed better in 10 cases. Flib outperforms NNMake in 31 of the 41 cases.

We generated 1,000 decoys for each of the proteins in our PDB-Representative set (S1 Table) using SAINT2 and Flib libraries. We compared our results to the results obtained by generating 1,000 decoys with NNMake libraries using SAINT2 (Fig 8). The Flib libraries generated accurate models (TM-Score > 0.5) for 12 of the 41 cases in our test set. The NNMake libraries generated accurate models for 8 of the 41 cases. Of the 13 cases for which accurate models were generated by either method, Flib libraries performed better in 10. Flib failed to generate a correct model in only one case where NNMake libraries produced an accurate result, whereas NNMake libraries failed to generate a correct model in 5 cases where Flib libraries produced good models.

## Discussion

In this work, we have established that removal of homologs from any fragment library generation pipeline is essential to ensure that the precision and coverage obtained are representative of a realistic *de novo* structure prediction scenario, otherwise overly promising results will be shown.

We tested different template databases (subsets of the PDB) in order to understand how database size and selectiveness can affect the quality of generated fragment libraries. Our analysis revealed that larger template databases give marginally better results. This implies that errors introduced by low quality structures are compensated for by the diversity introduced by using more proteins.

The correlation between sequence and secondary structure scores and fragment RMSD to the native structure was also investigated. We observed that, once homologs are excluded from template databases, sampling at random from fragments that satisfy a score cutoff produces better results than extracting fragments exhaustively. We opted to employ a combination of both methods (random sampling and exhaustive sampling) in Flib. Exhaustive extraction is useful for finding high scoring fragments that are likely to be good, whereas random methods increase the diversity of the final ensemble. We have observed that ranking fragments according to predicted torsion angles improved results. Previous results suggest that predicted torsion angles perform better than predicted secondary structure in assisting protein structure prediction [24]. Fragments extracted from protein threading hits were also added to our fragment libraries. These fragments improved the accuracy of generated libraries and these fragment ensembles become more consistent because a large number of fragments are extracted from the same template structure. It has been reported that fragment consistency might be more important than target RMSD to generate good models [5]. Fragment consistency is defined as how well a set of fragments representing different target positions can be pieced together.

Our analyses have also revealed a strong relationship between library fragment RMSD to the native structure and the predominant predicted secondary structure of the fragments. We have separated fragments into four distinct classes (SS Classes) based on their predominant predicted secondary structure and have shown that more lenient cutoffs lead to higher precision in *majority loop* and *other* fragments, but stricter cutoffs lead to higher precision in the *majority α-helical* and *majority β-strand* fragments. These results also suggest that it is harder to predict good fragments for positions that are represented by *majority loop* or *other* fragments. During model generation, it may be beneficial to concentrate sampling efforts into these harder to predict positions.

Flib presents a better balance between coverage and precision when compared against HHFrag and NNMake. Compared to NNMake, Flib can generate fragments with varying lengths. This has been previously shown to improve protein structure prediction. Flib fragments are, on average, 1 residue shorter than NNMake fragments. Considering that RMSD is correlated with fragment length, we investigated whether Flib's higher precision could be explained due to its shorter fragments. We built a new fragment library considering only the first eight residues of each of the fragments output by NNMake. We noticed a slight improvement in the precision of NNMake's libraries, but Flib libraries still presented higher precision and coverage. When compared to HHFrag, Flib presents a higher coverage. Flib also outputs fragments for every target position, which is necessary for structure prediction.

We have compared the improvement obtained by using Flib libraries against NNMake libraries in a protein structure prediction framework. Flib libraries generated accurate models in 12 out of the 41 test cases. Further, our libraries outperform NNMake in 10 of the 13 cases where an accurate model was generated.

The number of decoys we have generated during our analysis is comparable to the number of decoys that were generated in previous works [10,19]. However, this number is relatively low and it is hard to assess the statistical significance of our results. For that reason, we compared the RMSD to the native structure of the best fragment for each target position obtained by each of Flib, NNMake and HHFrag (S8 Fig). In principle, if the fragment assembly is exhaustive or has reached convergence, it is the best fragment within each window that ultimately determines the outcome of *de novo* structure prediction. Therefore, this comparison describes how well a fragment library can be used to model a target independent of the number of decoys generated. The RMSDs of the best fragments for each target position between Flib and NNMake are comparable and as Flib libraries are nearly 10 times smaller they are better suited for structure prediction. Furthermore, the modelling step in our analysis is computationally intensive. For that reason, we chose to work with a reduced number of targets (41 proteins). We believe that our data set is large enough to assess the impact of using better libraries in a structure prediction context, despite probably not being large enough to be representative of the complete protein fold space.

## Materials and Methods

### Training Data Set

Our fragment library generation method was trained using a set of 43 structurally diverse proteins extracted from the PDB [16]. A full list of these proteins is given in S2 Table. These proteins are all single chain, single domain proteins proportionally distributed into the four SCOP [26] protein classes: all alpha, all beta, alpha/beta, and alpha+beta. They are also evenly spread in terms of length, ranging from 50 to 500 residues. Each of the proteins in our dataset belongs to a different Pfam family [27]. Secondary structure for each protein was computed using the software DSSP [28] and predicted secondary structure was computed using PSIPRED [29]. Predicted torsion angles for each protein were computed using the software SPINE-X [24,25].

### PDB-Representative Validation Data Set

Our fragment library generation method was validated using a set of 41 structurally diverse proteins extracted from the PDB [16]. A full list of these proteins is given in S1 Table. These proteins are all single chain, single domain proteins proportionally distributed into the four SCOP [26] protein classes: all alpha, all beta, alpha/beta, and alpha+beta. They are also evenly spread in terms of length, ranging from 50 to 500 residues.

### CASP Validation Data Set

We have also validated Flib on a set of 275 domains that were used in CASP9 and CASP10. We have used all domains available from both experiments to compose this validation set.

### Homolog Identification

Sequence homologue identification was performed using HHSearch [11]. We have used HHSearch with default parameters: database = PDB70_05Jun14, number of iterations = 2, E-value cutoff for inclusion in resulting alignment = 0.001. HHSearch hits with a probability of 99.5% or higher were considered to be homologs.

### Template Databases

There are two main criteria used for culling protein structures from the PDB [16] when assembling template databases: pairwise sequence identity and resolution. NNMake accepts what it defines as non-identical sequences (50% identity cutoff) whereas HHFrag imposes a stricter cutoff of 25% pairwise sequence identity. NNmake only uses structure with a resolution better than 2.5Å, whereas HHFrag does not impose any resolution cutoff. We built three protein template databases by culling sequences from the PDB [16]: Database Flib, Database NNMake and Database HHFrag. For Database Flib, we removed any protein that presented a resolution worse than 5Å or that presented more than 90% sequence identity to another protein already in the database. For Database NNMake, we used the same selection criteria defined by NNMake (resolution cutoff of 2.5Å and 50% identity cutoff). Database HHFrag used the same criteria as HHFrag: the April, 2010 build of PDBselect25 [15].

These databases were further processed: we precomputed the secondary structure for every entry in each of the template databases using DSSP [28]. We classified each residue in all protein sequences into seven distinct groups based on their backbone torsion angles. These seven groups are based on areas of the Ramachandran Plot as defined by Choi *et al* [30]. These areas define the environments for our environment-specific substitution matrices (see Fragment Scores). Therefore, each entry in a database is represented by three strings: sequence, secondary structure and Ramachandran region identifier.

### Fragment Scores

Three main scores were built and tested. All of the scores are defined using a pairwise comparison between fragment and target residues.

Ramachandran-specific Sequence Score: The fragment Ramachandran score is defined as the sum of the score of each pair of fragment/target residues. We have defined environment-specific amino-acid substitution matrices to assign scores to a pair of residues [30]. These matrices have been built in a similar fashion to the BLOSUM matrices. They describe the propensity for an amino-acid substitution within a given environment and are extrapolated from amino-acid frequencies encountered in multiple sequence alignments. We defined the environments as the seven Ramachandran plot regions in [30]. This score incorporates additional torsion angle information compared to a standard sequence alignment score (i.e. using the BLOSUM62 scoring matrix).

Secondary Structure Score: this score is based on a pairwise comparison between the target fragments’ predicted secondary structures as output by PSIPRED [29] to the database fragments’ known secondary structure as output by DSSP. We used the following scoring scheme: Match = 2, Mismatch = -2.

Predicted Torsion Angle Score: the torsion angles (ϕ,ψ) for every database fragment was computed and compared to the predicted torsion angles for the target fragment as output by SPINE-X [24,25]. We define the predicted torsion angle score as the sum of the absolute differences between predicted and real ϕ angles and between predicted and real ψ angles for each fragment residue.

### Fragment Extraction with Flib

Fragments were generated for each of the proteins in our test data set using two extraction methods: random extraction and exhaustive extraction. In all cases, all fragments from homologs to the target were removed. We classified each target position according to its predicted predominant predicted secondary structure into four SS classes: *majority alpha-helix*, *majority beta-strand*, *majority loop* and *other*. For example, if more than half of the residues of a fragment are part of an alpha-helix, then the fragment is classified as a *majority alpha-helix* fragment. If a fragment does not have a predominant SS type, we place it in the *other* category.

The random extraction method consisted of scoring 5,000 randomly selected fragments of varying length per target sequence position from the template databases. The length of each fragment was randomized to be between six to 20 residues. Each fragment was scored according to the Ramachandran score and the Secondary Structure score. Every fragment is accepted depending on whether its score satisfies an acceptance cutoff. We have selected the cutoffs that achieve the best precision whilst maximising the coverage (S3 Fig). Different cutoffs were determined and used within each fragment SS class. The resulting library presents, on average, 2,000 fragments per target position.

In exhaustive extraction all possible fragments from a template database were scored against every position in the target. Analogous to the random extraction, fragments were scored based on the Ramachandran-specific Sequence Score and the predicted secondary structure score. The top 1,000 scoring fragments are selected for each target position as we found that the precision was not increased by the inclusion of more fragments.

The top 1,000 fragments per target position obtained by exhaustive extraction are merged with the 2,000 fragments per target position obtained by random extraction. The resulting fragment library presents approximately 3,000 fragments per target position (LIB3000).

For each target position, we rank the fragments in LIB3000 according to the predicted torsion angle score. We select the top 20 highest scoring fragments (LIB20) per target position according to the predicted Torsion angle score. We further enrich our final libraries by including any fragment from LIB3000 that presents less than 0.5 Å RMSD to the highest scoring fragment for a given position.

In the final step of our routine, we perform protein threading using the target sequence as input to HHSearch [17]. Default parameters for HHSearch were used to perform protein threading. Protein threading hits that originated from homologs, as described earlier, are removed from HHSearch's output. We extract every possible nine-residue fragment from the remaining threading hits (Protein Threading Library). The fragments in the Protein Threading library are ranked according to hit score output by HHSearch. We select a maximum of 20 fragments per target position. Fragments belonging to *majority alpha-helical* positions are removed from the Protein Threading Library in a post-processing step. All fragments in the Protein Threading Library are added to LIB20 to generate the final output of Flib. This final library presents, on average, ~33 fragments per target position.

### Validation

Two commonly used metrics to assess fragment library quality are global precision and coverage. Precision is defined as the number of good fragments divided by the total number of fragments in a library (the proportion of good fragments in the libraries). Coverage is defined as the number of residues represented by at least one good fragment divided by the number of residues of the target (the proportion of protein residues represented by a good fragment).

The quality of fragments was assessed by superimposing the fragment on to the target's known structure. We have varied the good fragment cutoff between 0.1 to 2.0 Å to compute a curve for precision and coverage. Fragments with an RMSD to the native structure below this varying cutoff are considered to be good fragments.

### HHFrag

In order to generate fragment libraries using HHFrag, we have used HHFrag v2.1 with default parameters.

### NNMake

We have used NNMake from the Build 3.5 of MiniROSETTA. In order to generate the fragment libraries, default parameters for NNMake were used.

### Model Generation

We have generated 1,000 decoys for every protein in our Validation set using two different approaches: Flib’s fragment libraries with SAINT2, NNMake's fragment libraries with SAINT2.

### SAINT2

There is evidence that suggests that co-translational aspects of protein folding could assist protein structure prediction [22, 31, 32, 33, 34]. SAINT2 is a co-translational protein structure prediction software programme [22]. It is a fragment-based approach that relies on sampling the conformational space in a sequential fashion. Unlike other fragment-based approaches, instead of starting with a fully elongated sequence, SAINT2 starts with a short peptide and moves from the heuristic routine are intercalated with an extrusion (a fragment replacement that happens at the end of the nascent chain and that elongates the peptide by one residue).

We have incorporated a correlated mutations potential into SAINT2 as it has been reported to improve modelling results [35, 36]. We have used PSICOV [35] to predict protein contacts for each target in our data set. PSICOV generated predictions for 34 of the 43 proteins in our data set (the accuracy of the contact predictions can be found in S3 Table). The predicted contact potential within SAINT2 was based on the contact potential described in [36].

## Supporting Information

S1 Fig Analysis of the precision of fragment libraries generated by Flib using three different template databases: database Flib (red), database NNMake (green), and database HHFrag (blue). We varied the RMSD to native structure cutoff to define a good fragment from 0.1 to 2.0 Angstroms (x-axis). The average precision for the LIB20 on the 43 proteins in the test data set is shown for each of the different template databases. The precision indicates the proportion of good fragments in the generated libraries (y-axis).
(TIF)

S2 Fig Plot of fragment RMSD to native structure against three different scores: Ramachandran sequence score (A), Predicted Secondary Structure Score (B), and Predicted Torsion Angle Score (C). Results are shown for 1,000 fragments extracted at random for each of the 43 proteins in our test data set.
(TIF)

S3 Fig Example of Sequence Score cutoff selection. We have evaluated the average precision and coverage (y-axis) of fragment libraries generated by the random extraction method on our test set of 43 proteins. We have varied the Ramachandran-Specific Sequence Score cutoff (x-axis) for accepting fragments in the library and assessed the effect of the cutoff on the precision (bars) and the coverage (red line) of generated libraries. We select the cutoff that maximises precision while maintaining coverage as close as possible to 100% (illustrated by the blue line).
(TIF)

S4 Fig Analysis of the impact of fragments extracted from protein threading hits on the precision within each SS class. Precision is shown for the fragment libraries generated by LIB20, Protein Threading Hits and Flib (a combination of the two previous approaches). We varied the RMSD to native structure cutoff to define a good fragment from 0.1 to 2.0 Angstroms (x-axis). The average precision within each SS Class on the 43 proteins in the test data set are shown.
(TIF)

S5 Fig Comparison between HHFrag, NNMake and Flib. Precision (left) and coverage (right) of fragment libraries generated using NNMake (red), HHFrag (green) and Flib (blue). The precision and coverage of the fragment libraries are averaged on a set of 275 protein domains that were used in CASP9 and CASP10. We varied the RMSD cutoff to define a good fragment (x axis) and evaluated the precision (proportion of good Fragments in the libraries) and coverage (proportion of protein residues represented by a good fragment) for each method.
(TIF)

S6 Fig Precision of fragment libraries generated using NNMake (red), HHFrag (green), and Flib (blue) separated by SS Class. The precision of the fragment libraries were averaged on a set of 275 protein domains that were used in CASP9 and CASP10. We varied the cutoff to define a good fragment (x axis) and evaluated the precision (proportion of good fragments in the libraries) for each method within four different SS classes: majority α-helical (top left), majority β-strand (top right), majority loop (bottom right) and other (bottom left).
(TIF)

S7 Fig Distribution of RMSDs to the native structure of all fragments as generated by NNMake (red) and Flib (blue). Fragments were generated for the 41 proteins in the PDB-Representative validation set.
(TIF)

S8 Fig Distribution of RMSDs to the native structure of the best fragment per target position as generated by each of NNMake (red), HHFrag (green) and Flib (blue). Fragment libraries were generated for the 41 proteins in our PDB-Representative validation set. Best fragments for each target position were selected using the RMSD to the native structure.
(TIF)

S1 Table The 41 proteins comprising our PDB-representative validation data set separated by SCOP classes. Proteins are single-domain, single chain, and belong to distinct PFam families.
(DOC)

S2 Table The 43 proteins comprising our test data set separated by SCOP classes. Proteins are single-domain, single chain, and belong to distinct PFam families.
(DOC)

S3 Table Accuracy of Contact Predictions as generated by PSICOV for our PDB-representative validation data set of 41 proteins. The PDB IDs of each protein are described in the first column. The second column describes the accuracy (true positives/total predictions). The number of contacts predicted correctly can be observed on the third column and the total number of predicted contacts can be observed in the fourth column.
(DOC)
