# Supplementary figures and images for: Building a Better Fragment Library for De Novo Protein Structure Prediction
Source: PLoS One. 2015 Apr 22;10(4):e0123998. doi: 10.1371/journal.pone.0123998 (PMC4406757; doi:10.1371/journal.pone.0123998)

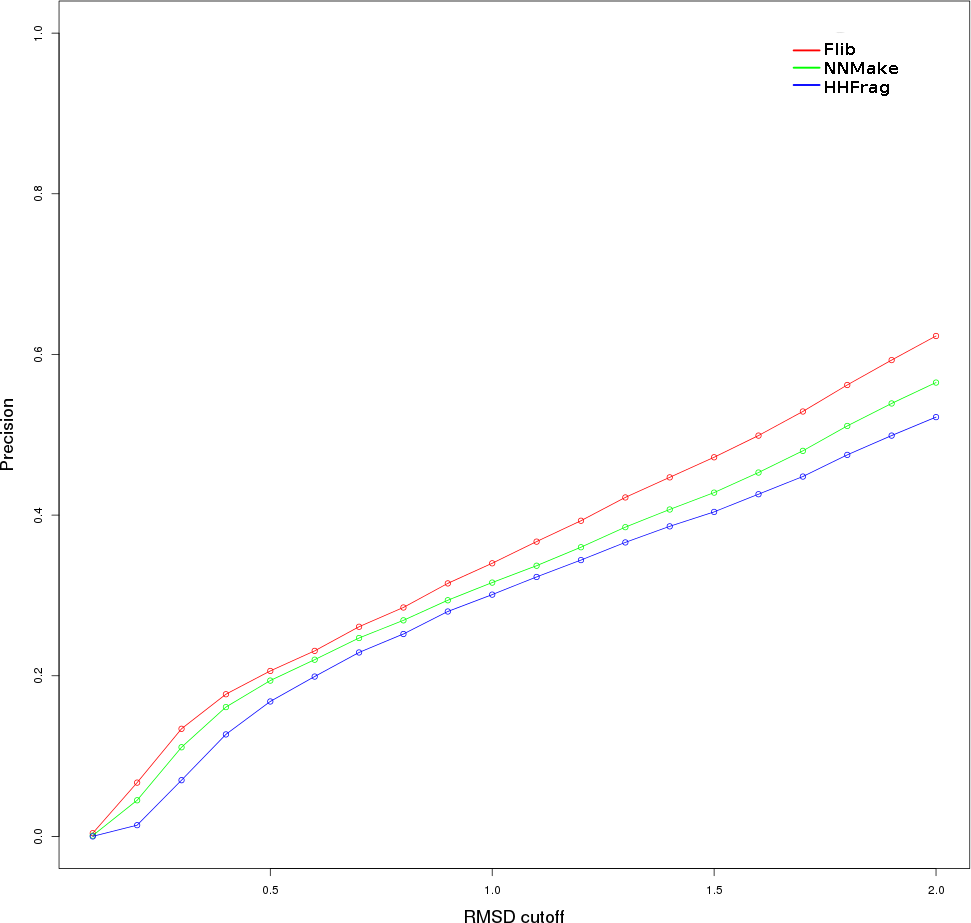

Supplement: S1 Fig — We varied the RMSD to native structure cutoff to define a good fragment from 0.1 to 2.0 Angstroms (x-axis). The average precision for the LIB20 on the 43 proteins in the test data set is shown for each of the different template databases. The precision indicates the proportion of good fragments in the generated libraries (y-axis). (TIF) [file pone.0123998.s001.tif]

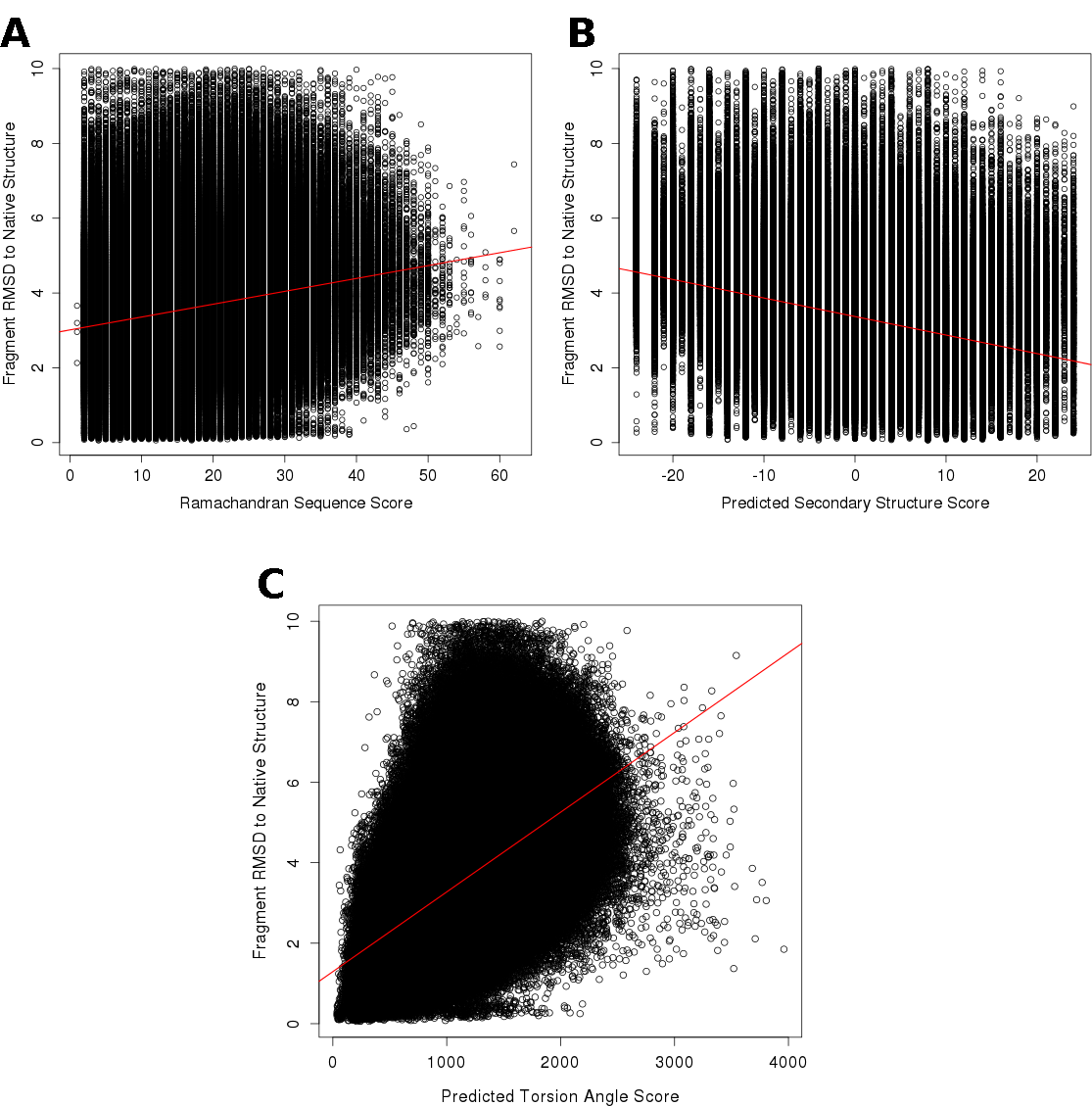

Supplement: S2 Fig — Results are shown for 1,000 fragments extracted at random for each of the 43 proteins in our test data set. (TIF) [file pone.0123998.s002.tif]

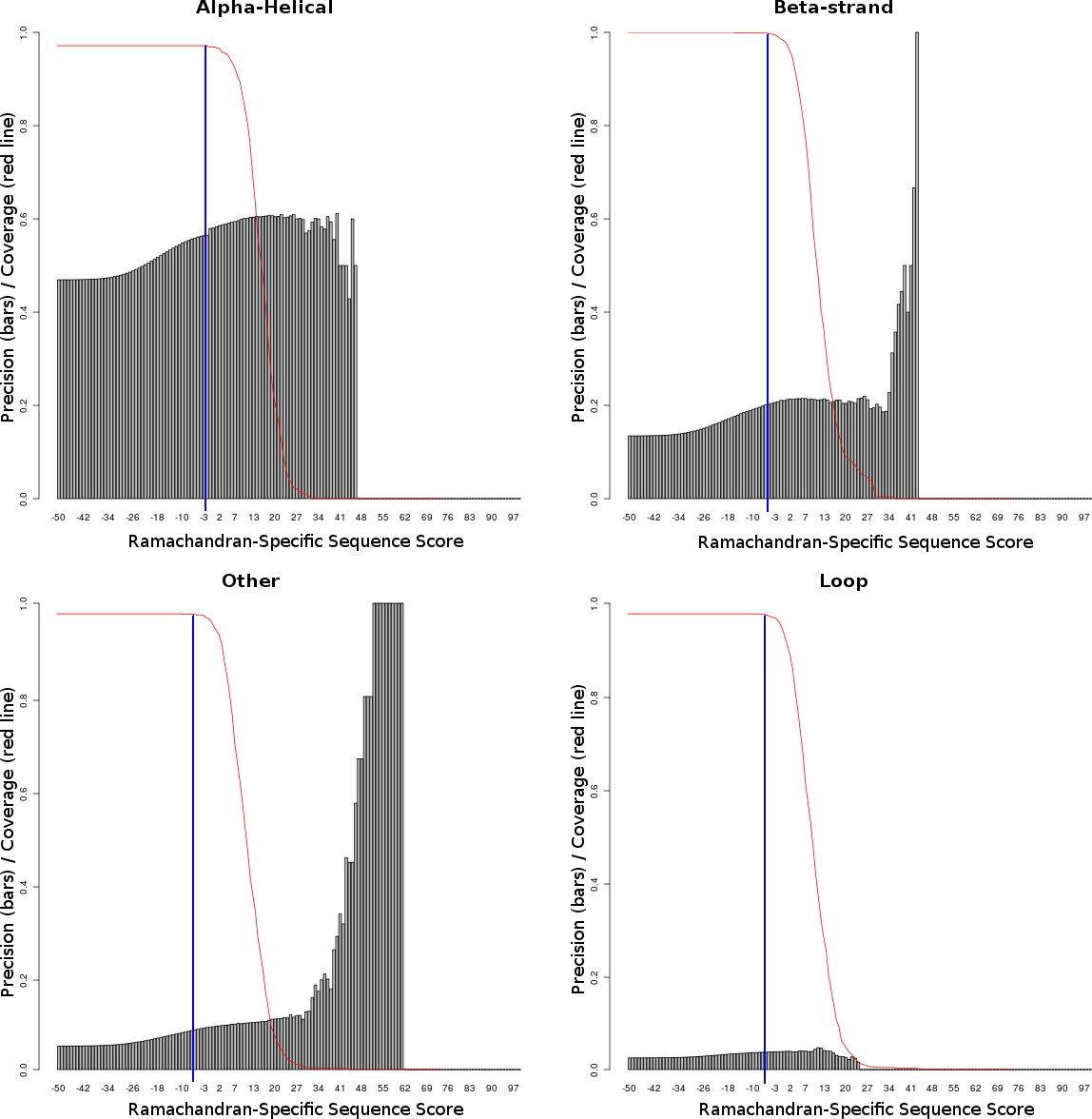

Supplement: S3 Fig — We have evaluated the average precision and coverage (y-axis) of fragment libraries generated by the random extraction method on our test set of 43 proteins. We have varied the Ramachandran-Specific Sequence Score cutoff (x-axis) for accepting fragments in the library and assessed the effect of the cutoff on the precision (bars) and the coverage (red line) of generated libraries. We select the cutoff that maximises precision while maintaining coverage as close as possible to 100% (illustrated by the blue line). (TIF) [file pone.0123998.s003.tif]

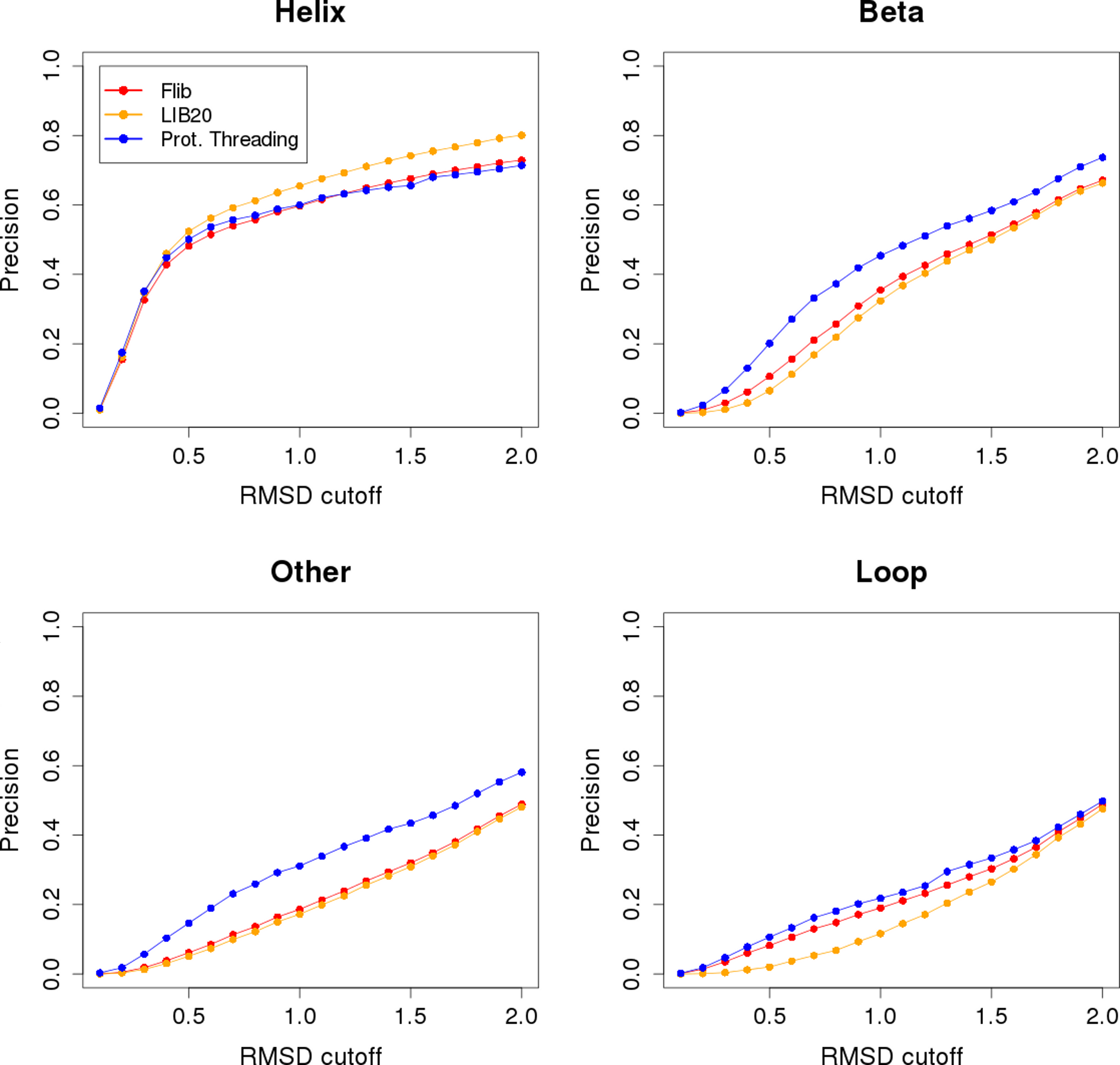

Supplement: S4 Fig — Precision is shown for the fragment libraries generated by LIB20, Protein Threading Hits and Flib (a combination of the two previous approaches). We varied the RMSD to native structure cutoff to define a good fragment from 0.1 to 2.0 Angstroms (x-axis). The average precision within each SS Class on the 43 proteins in the test data set are shown. (TIF) [file pone.0123998.s004.tif]

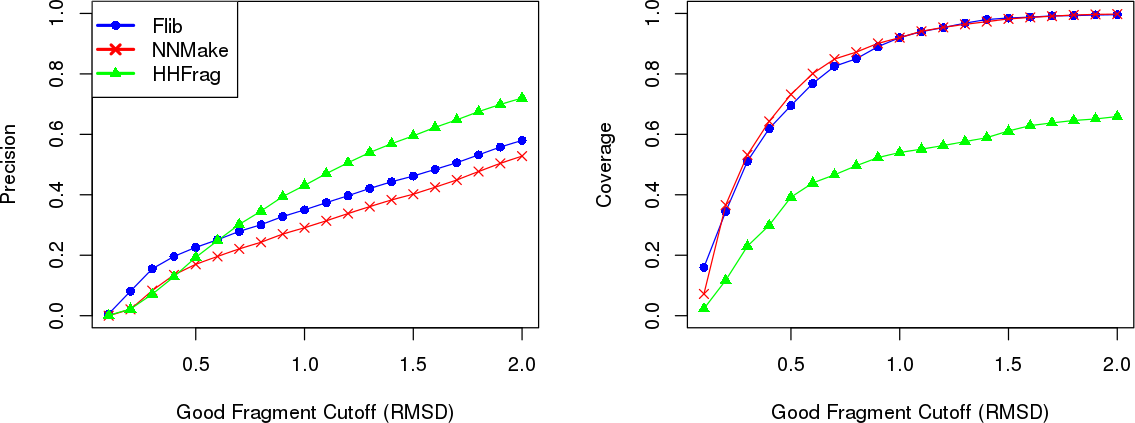

Supplement: S5 Fig — Precision (left) and coverage (right) of fragment libraries generated using NNMake (red), HHFrag (green) and Flib (blue). The precision and coverage of the fragment libraries are averaged on a set of 275 protein domains that were used in CASP9 and CASP10. We varied the RMSD cutoff to define a good fragment (x axis) and evaluated the precision (proportion of good Fragments in the libraries) and coverage (proportion of protein residues represented by a good fragment) for each method. (TIF) [file pone.0123998.s005.tif]

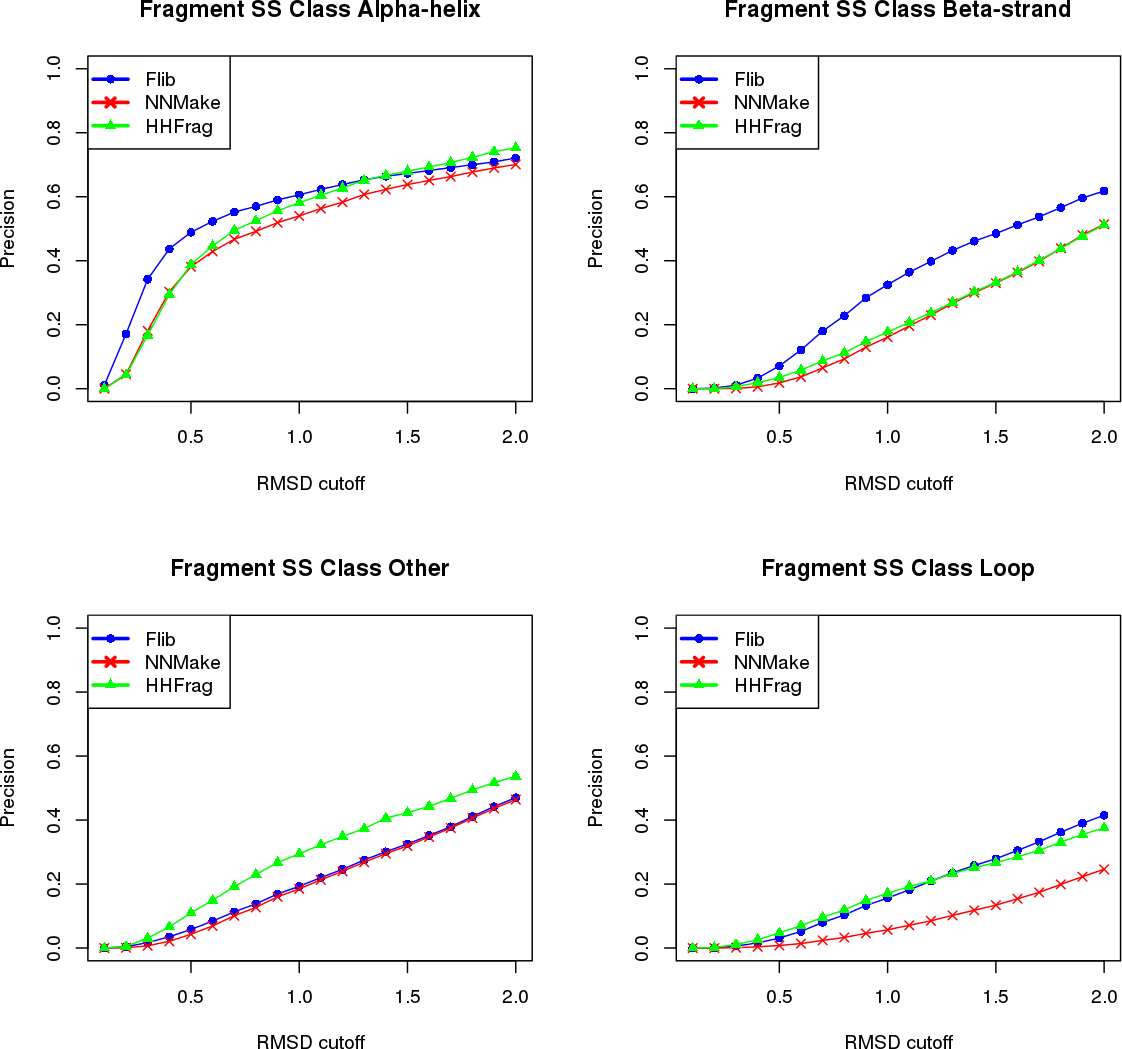

Supplement: S6 Fig — The precision of the fragment libraries were averaged on a set of 275 protein domains that were used in CASP9 and CASP10. We varied the cutoff to define a good fragment (x axis) and evaluated the precision (proportion of good fragments in the libraries) for each method within four different SS classes: majority α-helical (top left), majority β-strand (top right), majority loop (bottom right) and other (bottom left). (TIF) [file pone.0123998.s006.tif]

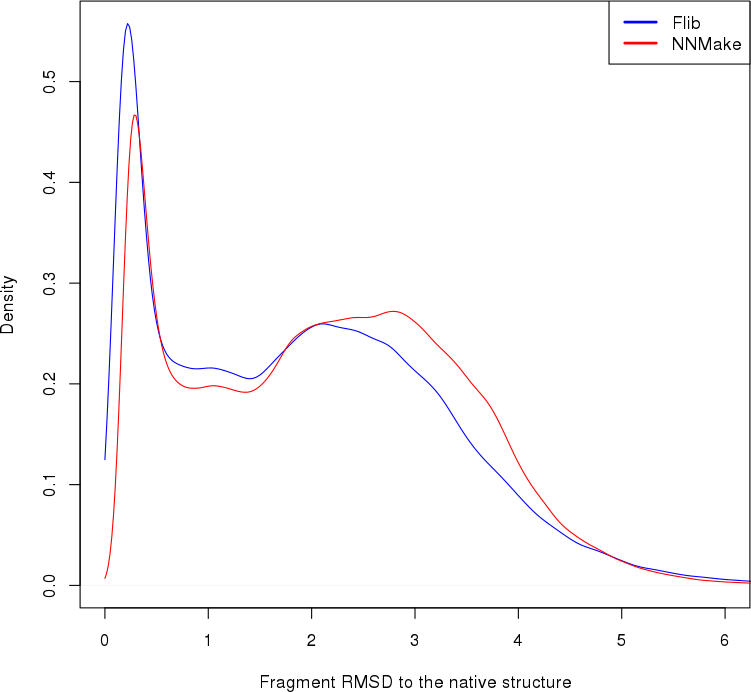

Supplement: S7 Fig — Fragments were generated for the 41 proteins in the PDB-Representative validation set. (TIF) [file pone.0123998.s007.tif]

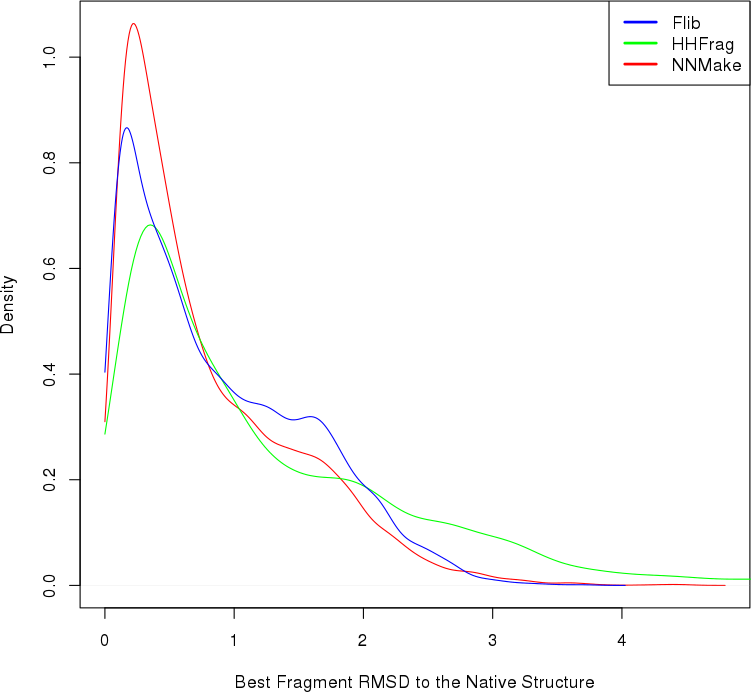

Supplement: S8 Fig — Fragment libraries were generated for the 41 proteins in our PDB-Representative validation set. Best fragments for each target position were selected using the RMSD to the native structure. (TIF) [file pone.0123998.s008.tif]
