# Supplementary material for: Building a Better Fragment Library for De Novo Protein Structure Prediction
Source: PLoS One. 2015 Apr 22;10(4):e0123998. doi: 10.1371/journal.pone.0123998 (PMC4406757; doi:10.1371/journal.pone.0123998)
Supplement: S1 Table — Proteins are single-domain, single chain, and belong to distinct PFam families. (DOC) [file pone.0123998.s009.doc]

**S1 Table. The 41 proteins comprising our PDB-Representative validation data set separated by SCOP classes.** Proteins are single-domain, single chain, and belong to distinct PFam families.

|  | **SCOP Class α / β** | | | |
| --- | --- | --- | --- | --- |
|  | **PDB ID** | **Pfam Family ID** | **Protein Length** | **Resolution** |
|  | 1AIU | PF00085 | 105 Residues | 2.00 A |
|  | 1NAT | PF00072 | 124 Residues | 2.45 A |
|  | 2RN2 | PF00075 | 155 Residues | 1.48 A |
|  | 1ILW | PF00857 | 180 Residues | 2.05 A |
|  | 1VL1 | PF01182 | 232 Residues | 1.55 A |
|  | 1XWY | PF01026 | 264 Residues | 2.00 A |
|  | 2HVM | PF00704 | 273 Residues | 1.80 A |
|  | 1OBR | PF00246 | 326 Residues | 2.3 A |
|  | 1VFF | PF00232 | 423 Residues | 2.50 A |
|  | 1SMD | PF00128 | 496 Residues | 1.6 A |
|  | **SCOP Class α + β** | | | |
|  | **PDB ID** | **Pfam Family ID** | **Protein Length** | **Resolution** |
|  | 1WM3 | PF00240 | 72 Residues | 1.2 A |
|  | 1CEW | PF00031 | 107 Residues | 2.0 A |
|  | 1EKG | PF01491 | 127 Residues | 1.8 A |
|  | 1Z2U | PF00179 | 150 Residues | 1.1 A |
|  | 1SQW | PF03657 | 188 Residues | 1.90 A |
|  | 1XKR | PF04509 | 203 Residues | 1.75 A |
|  | 1W66 | PF03099 | 232 Residues | 1.08 A |
|  | 1RL0 | PF00161 | 255 Residues | 1.4 A |
|  | 2YVT | PF12850 | 260 Residues | 1.60 A |
|  | 1MSK | PF02965 | 331 Residues | 1.8 A |
|  | 1AYE | PF02244 | 401 Residues | 1.8 A |
|  | 1B4V | PF00732 | 504 Residues | 1.5 A |
|  | **SCOP Class All β** | | | |
|  | **PDB ID** | **Pfam Family ID** | **Protein Length** | **Resolution** |
|  | 1CSP | PF00313 | 67 Residues | 2.45 A |
|  | 1BMG | PF13895 | 98 Residues | 2.5 A |
|  | 1XD6 | PF01453 | 112 Residues | 2.0 A |
|  | 1NEP | PF02221 | 130 Residues | 1.7 A |
|  | 1CZT | PF00754 | 160 Residues | 1.87 A |
|  | 1T9F | PF02815 | 187 Residues | 2.00 A |
|  | 2AYH | PF00722 | 214 Residues | 1.6 A |
|  | 1P6F | PF13895 | 241 Residues | 2.2 A |
|  | 1SEF | PF07883 | 274 Residues | 2.05 A |
|  | 1WL7 | PF04616 | 312 Residues | 1.9 A |
|  | 1OKQ | PF00054 | 394 Residues | 2.80 A |
|  | **SCOP Class All α** | | | |
|  | **PDB ID** | **Pfam Family ID** | **Protein Length** | **Resolution** |
|  | 1ENH | PF00046 | 54 Residues | 2.1 A |
|  | 2J9V | PF03997 | 99 Residues | 2.0 A |
|  | 2MHR | PF01814 | 118 Residues | 1.3 A |
|  | 1JWF | PF00790 | 147 Residues | 2.10 A |
|  | 1SFE | PF01035 | 180 Residues | 2.10 A |
|  | 1SDI | PF04356 | 213 Residues | 1.65 A |
|  | 1VIN | PF00134 | 268 Residues | 2.0 A |
|  | 1V5C | PF01270 | 386 Residues | 2.0 A |
