# Supplementary material for: Building a Better Fragment Library for De Novo Protein Structure Prediction
Source: PLoS One. 2015 Apr 22;10(4):e0123998. doi: 10.1371/journal.pone.0123998 (PMC4406757; doi:10.1371/journal.pone.0123998)
Supplement: S2 Table — Proteins are single-domain, single chain, and belong to distinct PFam families. (DOC) [file pone.0123998.s010.doc]

**S2 Table. The 43 proteins comprising our test data set separated by SCOP classes.** Proteins are single-domain, single chain, and belong to distinct PFam families.

|  | **SCOP Class α / β** | | | |
| --- | --- | --- | --- | --- |
|  | **PDB ID** | **Pfam Family ID** | **Protein Length** | **Resolution** |
|  | 1H75 | PF00462 | 81 Residues | 1.70 A |
|  | 1IU9 | PF01177 | 111 Residues | 2.04 A |
|  | 1E6K | PF08924 | 130 Residues | 2.00 A |
|  | 1P90 | PF02579 | 145 Residues | 1.80 A |
|  | 1FTG | PF00258 | 168 Residues | 2.00 A |
|  | 1QCY | PF00092 | 193 Residues | 2.30 A |
|  | 2A14 | PF00072 | 263 Residues | 1.70 A |
|  | 1IZZ | PF01965 | 283 Residues | 2.31 A |
|  | 1QUE | PF00175/PF00970 | 303 Residues | 1.8 A |
|  | 1KRM | PF00962 | 356 Residues | 2.5 A |
|  | 3BSG | PF07821 | 414 Residues | 1.95 A |
|  | 1PGN | PF00393 | 482 Residues | 2.3 A |
|  | **SCOP Class α + β** | | | |
|  | **PDB ID** | **Pfam Family ID** | **Protein Length** | **Resolution** |
|  | 1VJW | PF00037 | 60 Residues | 1.75 A |
|  | 1MWP | PF02177 | 96 Residues | 1.8 A |
|  | 1GNU | PF02991 | 117 Residues | 1.75 A |
|  | 1R9H | PF00254 | 135 Residues | 1.80 A |
|  | 206L | PF00959 | 164 Residues | 1.75 A |
|  | 2FS3 | PF13302 | 195 Residues | 1.52 A |
|  | 1DZF | PF01191 | 215 Residues | 1.9 A |
|  | 1DXJ | PF00182 | 242 Residues | 1.8 A |
|  | 1MAT | PF00557 | 264 Residues | 2.4 A |
|  | 1JKS | PF01163 | 294 Residues | 1.50 A |
|  | 1MC4 | PF01118 | 370 Residues | 2.77 A |
|  | 2FKF | PF02879 | 462 Residues | 2.00 A |
|  | **SCOP Class All β** | | | |
|  | **PDB ID** | **Pfam Family ID** | **Protein Length** | **Resolution** |
|  | 1MHN | PF06003 | 59 Residues | 1.8 A |
|  | 1TEN | PF00041 | 90 Residues | 1.8 A |
|  | 2G1L | PF00498 | 104 Residues | 2.6 A |
|  | 1IFR | PF00932 | 121 Residues | 1.4 A |
|  | 1BFG | PF00167 | 146 Residues | 1.6 A |
|  | 2FR2 | PF08768 | 172 Residues | 1.5 A |
|  | 1EE6 | PF03211 | 197 Residues | 2.30 A |
|  | 1UAI | PF08787 | 224 Residues | 1.2 A |
|  | 2C9A | PF00047 | 259 Residues | 2.7 A |
|  | 1O4Y | PF00722 | 288 Residues | 1.48 A |
|  | 1HG8 | PF00295 | 349 Residues | 1.73 A |
|  | 1NKG | PF09284 | 508 Residues | 1.50 A |
|  | **SCOP Class All α** | | | |
|  | **PDB ID** | **Pfam Family ID** | **Protein Length** | **Resolution** |
|  | 1AIL | PF00600 | 73 Residues | 1.9 A |
|  | 1RRO | PF13202 | 108 Residues | 1.3 A |
|  | 1U61 | PF00636 | 138 Residues | 2.15 A |
|  | 1SL8 | PF13202 | 191 Residues | 1.70 A |
|  | 1QUU | PF00435 | 250 Residues | 2.5 A |
|  | 1T5J | PF03747 | 313 Residues | 2.7 A |
|  | 1PO5 | PF00067 | 476 Residues | 1.6 A |
