# Supplementary material for: Building a Better Fragment Library for De Novo Protein Structure Prediction
Source: PLoS One. 2015 Apr 22;10(4):e0123998. doi: 10.1371/journal.pone.0123998 (PMC4406757; doi:10.1371/journal.pone.0123998)
Supplement: S3 Table — The PDB IDs of each protein are described in the first column. The second column describes the accuracy (true positives/total predictions). The number of contacts predicted correctly can be observed on the third column and the total number of predicted contacts can be observed in the fourth column. (DOC) [file pone.0123998.s011.doc]

| **PDB ID** | **Accuracy (%)** | **Correct Predictions** | **Total Predictions** |
| --- | --- | --- | --- |
| 1BMG | - | 0 | 0 |
| 1SQW | - | 0 | 0 |
| 1V5C | - | 0 | 0 |
| 1VFF | - | 0 | 0 |
| 1XKR | - | 0 | 0 |
| 2J9V | - | 0 | 0 |
| 2YVT | 0.0 | 0 | 0 |
| 1W66 | 8.33 | 2 | 24 |
| 1T9F | 18.18 | 10 | 55 |
| 1SDI | 20.00 | 10 | 50 |
| 2MHR | 22.73 | 5 | 22 |
| 1RL0 | 22.92 | 11 | 48 |
| 1B4V | 27.78 | 30 | 108 |
| 1OKQ | 29.41 | 5 | 17 |
| 1SMD | 30.28 | 66 | 218 |
| 1NEP | 30.77 | 8 | 26 |
| 2HVM | 32.86 | 46 | 140 |
| 1JWF | 33.33 | 7 | 21 |
| 1EKG | 39.13 | 9 | 23 |
| 1WL7 | 48.25 | 69 | 143 |
| 1OBR | 49.70 | 82 | 165 |
| 1VIN | 51.43 | 18 | 35 |
| 1XD6 | 52.00 | 13 | 25 |
| 2AYH | 52.24 | 35 | 67 |
| 1MSK | 52.50 | 21 | 40 |
| 1VL1 | 54.05 | 60 | 111 |
| 1SFE | 55.26 | 21 | 38 |
| 1AYE | 55.56 | 5 | 9 |
| 1Z2U | 56.52 | 26 | 46 |
| 1CEW | 60.00 | 9 | 15 |
| 1XWY | 61.82 | 136 | 220 |
| 1ENH | 63.64 | 7 | 11 |
| 1AIU | 67.27 | 37 | 55 |
| 1CZT | 69.05 | 29 | 42 |
| 1ILW | 69.79 | 67 | 96 |
| 1WM3 | 70.59 | 12 | 17 |
| 2RN2 | 72.88 | 43 | 59 |
| 1CSP | 77.78 | 14 | 18 |
| 1P6F | 81.82 | 18 | 22 |
| 1NAT | 82.35 | 56 | 68 |
| 1SEF | 91.67 | 22 | 24 |

**S3 Table. Accuracy of Contact Predictions as generated by PSICOV for our PDB-Representative validation set of 41 proteins.** The PDB IDs of each protein are described in the first column. The second column describes the accuracy (true positives/total predictions). The number of contacts predicted correctly can be observed on the third column and the total number of predicted contacts can be observed in the fourth column.
